# The *Lsktm1* Locus Modulates Lung and Skin Tumorigenesis in the Mouse

**DOI:** 10.1534/g3.112.003525

**Published:** 2012-09-01

**Authors:** Antonella Galvan, Francesca Colombo, Sara Noci, Simonetta Pazzaglia, Mariateresa Mancuso, Giacomo Manenti, Karl W. Broman, Anna Saran, Tommaso A. Dragani

**Affiliations:** * Department of Predictive and Preventive Medicine; Fondazione IRCCS Istituto Nazionale dei Tumori, Milan, Italy; † Laboratory of Radiation Biology and Biomedicine, Agenzia Nazionale per le Nuove Tecnologie, l’Energia e lo Sviluppo Economico Sostenibile (ENEA) CR-Casaccia, Rome, Italy, and; ‡ Department of Biostatistics and Medical Informatics, University of Wisconsin, Madison, Wisconsin 53706

**Keywords:** disease models, cancer modifier genes, lung cancer, quantitative trait loci (QTLs), skin cancer, single-nucleotide polymorphisms

## Abstract

Alleles derived from skin tumor−resistant Car-R mice provide resistance to both skin and lung tumorigenesis over the susceptibility of the SWR/J strain. In an effort to map tumor modifier loci affecting both tumor types, we carried out a genetic linkage analysis in backcross SWR/J x (SWR/J x Car-R) mice and identified a locus (*Lsktm1*) on chromosome 1 linked to both skin (LOD score = 3.93) and lung (LOD score = 8.74) tumorigenesis. Two genes, *Igfbp5* and *Igfbp2*, residing in this locus and belonging to the insulin-like growth factor binding protein family were expressed at significantly greater levels in normal lung tissue from cancer-resistant Car-R mice than in cancer-susceptible SWR/J mice. Overexpression of the recombinant *Igfbp5* and *Igfbp2* genes in two lung cancer cell lines significantly inhibited clonogenicity (*P* < 0.0001). Collectively, we have identified a single polymorphic locus that affects skin and lung tumorigenesis and identify *Igfbp5* and *Igfbp2* as candidate modifier genes of lung tumorigenesis.

Deciphering the genetic basis of tumor susceptibility has been facilitated during the past decade by the development of strains of mice that markedly differ in their responses to carcinogens. One such line is the carcinogenesis-resistant (Car-R) mouse. This outbred line, derived from the crossing of eight inbred lines, was phenotypically selected for resistance to two-stage chemical induction of skin cancer (Saran *et al.* 2000). Car-R mice carry cancer resistance loci that protect from the development of not only skin papillomas but also lung surface tumors (Saran *et al.* 2002). In particular, when Car-R males were crossed with inbred SWR/J females, which are susceptible to skin and lung cancer, their F1 progeny retained resistance to both tumors, measured quantitatively as reduced incidence and lower tumor multiplicity. In that work, cancer was induced by a two-step chemical treatment, with tumor initiation by intraperitoneal injection of urethane, a carcinogen that induces both skin and lung cancer, and with promotion by topical application of 12-O-tetradecanoylphorbol-13-acetate (TPA), which causes skin cancer. The fact that alleles from the Car-R genome inhibited both skin and lung tumors suggests that these mice carry one or more loci that modulate both tumorigenesis processes.

To fine map the genetic loci underlying quantitative traits (QTL) like tumor multiplicity, backcross populations typically are used because if extreme phenotypic differences exist between the parental strains and dominant interactions are expected, they allow the segregation of cancer modifier loci in a shared genomic background (Darvasi 1998). In addition, if one of the parental lines used to generate the backcross population is outbred, the high number of recombinations provides a level of resolution for QTL mapping that is not attainable by normal linkage analysis (Balmain 2002). Therefore, because Car-R mice are highly genetically different from the SWR/J line and are dominantly resistant to chemically induced skin and lung carcinogenesis, genetic analysis of the SWR/J x (SWR/J x Car-R) backcross population initially described in 2002 (Saran *et al.* 2002) should lead to the identification of genetic components providing resistance to skin and lung cancer phenotypes, segregated in specific small genomic regions. That a single chromosomal region may be involved in the susceptibility to different tumors was previously hypothesized based on the observed clustering of loci affecting different tumor types in the mouse (Dragani 2003).

Here, we report the results of a genome-wide genetic analysis of this backcross population using a high-density single-nucleotide polymorphism (SNP) array. Analysis for linkage with tumor multiplicity identified a region of chromosome 1, which we have called *Lsktm1* locus, associated with both types of tumorigenesis. Within this locus, we identified *Igfbp5* and *Igfbp2* as the best candidate cancer modifier genes, members of the insulin-like growth factor binding protein family.

## Materials and Methods

### Ethics statement

Animal experiments were approved by the Institutional Animal Care and Use Committee of ENEA Laboratory and were conducted according to relevant national and international guidelines.

### Mice and nucleic acids

The pedigree used in the study consisted of two outbred Car-R male and 6 SWR/J female grandparents, 16 F1 sires, 16 SWR/J dams, and 231 male and female SWR/J x (SWR/J x Car-R) backcross mice. Results of phenotypic analysis of the first 148 backcross mice were reported (Saran *et al.* 2002) before expansion of the population. As described previously, the backcross mice that were 4 weeks of age were treated intraperitoneally with urethane (tumor initiation) and then, starting 7 days later, with topically applied TPA twice weekly for 14 weeks (tumor promotion). The number of skin papillomas was recorded at the end of experiment (30 weeks after tumor initiation). At 35 weeks of age, mice were killed, tumors on the lung surface were counted, and spleens were frozen.

Genomic DNA was extracted from frozen spleens of all the backcross mice and the founders of the pedigree. Total RNA was extracted from normal lung tissue of Car-R and SWR/J mice with RNeasy Midi Kit (QIAGEN, Valencia, CA), purified with RNeasy MinElute CleanUP Kit (QIAGEN), and checked for integrity by microcapillary electrophoresis (Bioanalyzer; Agilent Technologies, Santa Clara, CA). RNA was reverse-transcribed using ThermoScript RT-PCR system (Invitrogen, Carlsbad, CA).

### SNP genotyping

Mouse DNA was SNP genotyped using the GoldenGate assay according to the manufacturer’s protocol (Illumina, San Diego, CA), as described in (Vorraro *et al.* 2010).

### Expression microarray analysis

Samples of total RNA from normal lung tissue of 15 Car-R and 13 SWR/J untreated adult male mice were grouped in four pools per mouse strain, each pool deriving from 3-4 mice. Pooled total RNA (300 ng) was reverse-transcribed, labeled with biotin, and amplified overnight using the RNA TotalPrep Amplification kit (Ambion) according to the manufacturer’s protocol. Biotinylated cRNA was hybridized to MouseRef-8 v2.0 Expression BeadChips (Illumina). The array represents more than 24,000 bead types, each with a unique sequence derived from mouse genes in the National Centre for Biotechnology Information Reference Sequence and UniGene Database. Array chips were scanned with the Illumina BeadArray Reader, and primary data were collected using the supplied scanner software, resulting in a matrix containing 9,192 transcripts (*P* < 0.05, cutoff value to filter reliable genes).

### Real-time quantitative polymerase chain reaction (qPCR)

Quantitative mRNA levels were analyzed by real-time qPCR using the TaqMan Gene Expression Assay (Applied Biosystems) for *Igfbp2* (Mm00492632_m1) and *Igfbp5* (Mm00516037_m1). The real-time PCR amplification mixture contained 1 µL of cDNA template, 10 µL of 2X TaqMan Universal PCR Master Mix No AmpErase UNG (Applied Biosystems), and 1 µL of 20X TaqMan Gene Expression Assay Mix (Applied Biosystems) in a final volume of 20 µL. The mouse hypoxanthine guanine phosphoribosyl transferase 1 (*Hprt1*) gene (Mm00446968_m1) was used as internal control. Relative changes in mRNA levels were assessed using the comparative cycle threshold method, and relative quantities were calculated using as calibrator a pool of RNA from normal tissues.

### Clonogenic assay

*Igfbp5* and *Igfbp2* coding regions were PCR-amplified and cloned in pEF6/V5-His TOPO vector (Invitrogen; primers *Igfbp5*: 5′-agccagactccgagaaaatg-3′ and 5′-ctcaacgttactgctgtcgaag-3′; *Igfbp2*: 5′-ttgcccacaagccaacatg-3′ and 5′-ctgcacactttgggcatg-3′). Two lung cancer cell lines, purchased from American Type Culture Collection (Rockville, MD) and displaying a histotype of either human squamous cell carcinoma (NCI-H520) or mouse lung adenoma (LA-4) were transfected with recombinant or control empty vectors using FuGENE HD Transfection Reagent (Roche Applied Science, Basel, Switzerland). NCI-H520− and LA-4−transfected clones were selected with 2.0 and 2.5 µg/mL blasticidin (Invitrogen), respectively, for 2 weeks, methanol-fixed, stained with 10% Giemsa, and automatically counted with the Quantity One software (Bio-Rad Laboratories, Hercules, CA) or UVIDoc software (UVItec Limited, Cambridge, UK).

### Western blotting

Total protein was extracted with RIPA buffer (Pierce, Rockford, IL) from transfected NCI-H520 cells, resolved by SDS-polyacrylamide gel electrophoresis, and immunoblotted with anti-V5-HRP antibody (Invitrogen). The ECL SuperSignal West Pico Chemiluminescent Substrate system (Pierce) was used for detection.

### Statistical analysis

Lung and skin tumor multiplicity data were square-root transformed to improve the normality of their distributions. The association between phenotypes was analyzed using Pearson’s correlation and gender differences in phenotypes were analyzed by Welch *t*-test.

Interval mapping analysis was carried out using R/qtl (Broman *et al.* 2003), an add-on package to the general statistical software, R. LOD scores were considered significant if greater than the 95% LOD threshold, calculated by 1000 permutations. Sex and sibship identifiers were included as additive covariates; QTL x sex interactions were also explored as well as separate analyses of the two sexes.

Gene expression analyses were performed using BRB-ArrayTools developed by Dr. Richard Simon and BRB-ArrayTools Development Team (http://linus.nci.nih.gov/BRB-ArrayTools.html). Real-time quantitative PCR results and clonogenic data were tested by analysis of variance (ANOVA).

## Results

### A skin and lung tumor modifier locus maps on chromosome 1

The SWR/J x (SWR/J x Car-R) backcross population, originating from two Car-R grandsires, was first reported in 2002 (Saran *et al.* 2002) but was subsequently expanded to increase the number of animals analyzed from 148 to 231; this larger population allowed us to consider the impact of gender on tumor development. Phenotypic analysis after two-step tumor induction with urethane and TPA revealed, in the larger population, a significant sex effect for lung tumor multiplicity (Nlung), with males developing 1.3-fold more tumors than females (mean ± SE, n; males, Nlung = 23.5 ± 1.5, n = 129; females, Nlung = 17.7 ± 1.3, n = 98; *P* = 0.003, Welch *t*-test), whereas no sex effect was seen for skin tumor multiplicity (mean skin tumor multiplicity in all mice = 3.0). In agreement with previous findings (Saran *et al.* 2002), lung tumor multiplicity and skin papilloma multiplicity had a weak but significant correlation (Pearson’s coefficient = 0.20, *P* = 0.003), supporting the idea that a subset of loci may control both lung and skin tumor phenotypes.

To identify QTL affecting skin and lung tumor phenotypes in Car-R and SWR/J mice, we genetically analyzed a pedigree consisting of 210 of the backcross mice (two sibships were eliminated from analysis since their F1 sire had not been recorded), their 16 F1 male parents, the 16 SWR/J female inbred parents, and the 2 Car-R grandsires. Genome-wide SNP genotyping by microarray analysis produced a total of 1753 genotype calls per sample which, after filtering for informative data, provided 1017 SNPs for study. Because no differences were detected in the effects of the 4 Car-R alleles derived from the two Car-R grandparents, we assumed that the 4 Car-R alleles had a constant effect. Moreover, due to the observed gender effects on tumor multiplicity, genetic linkage was analyzed taking into account the interaction of sex.

**Figure 1:**
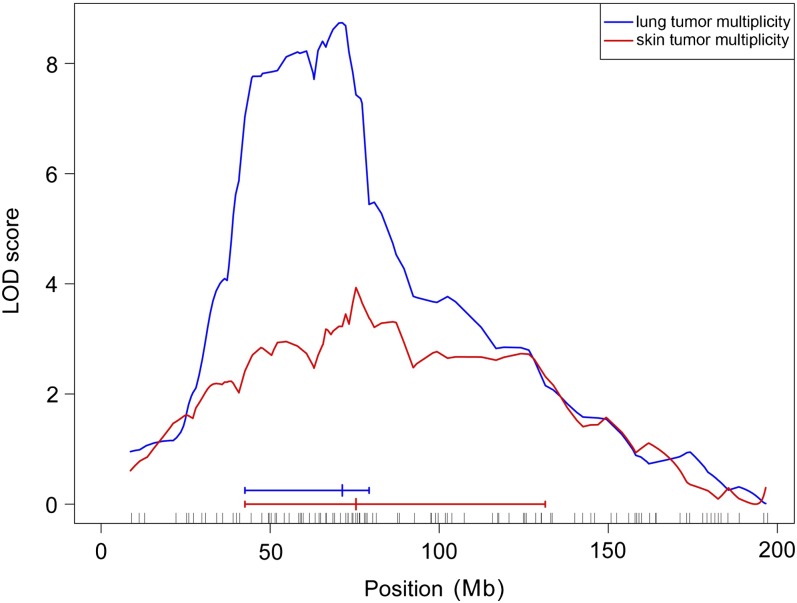
Interval mapping analysis of chromosome 1 region for skin and lung tumor susceptibility in SWR/J x (SWR/J x Car-R) mice. LOD curves for lung tumor multiplicity and skin tumor multiplicity are shown, with 1.5-LOD support intervals at the bottom. Map positions of SNP markers along the chromosome are indicated by small vertical lines.

By interval mapping analysis, we observed a significant linkage on chromosome 1 for both lung and skin tumor multiplicity (Figure 1). The peak LOD score for lung tumor multiplicity was 8.74, greater than the threshold for significant linkage (95% LOD threshold = 3.19). The peak was located near marker rs6312657 at 69.05 Mb (NCBI m37 assembly) whereas the 1.5-LOD support interval spanned from marker rs32779838 mapping at 41.15 Mb to rs13475931 at 76.37 Mb. For skin tumor multiplicity, the peak LOD score of 3.93 (above the 95% LOD threshold of 3.17) was located near marker rs4138000 at 75.83 Mb; the 1.5-LOD support interval spanned from rs32779838 mapping at 41.15 Mb to rs13464873 at 130.15 Mb. The 1.5-LOD support intervals of the two LOD score curves overlapped considerably, suggesting that a single locus controls both tumor phenotypes. This locus was designated “lung and skin tumorigenesis modifier 1” (*Lsktm1*).

The estimated variance explained by this chromosome 1 QTL was 6.3% and 5.9% for lung and skin tumor phenotypes, respectively. For both phenotypes, heterozygous mice carrying the Car-R alleles had fewer tumors than did mice that were homozygous for the SWR/J alleles because of the tumor inhibitory effects of Car-R−derived alleles for cancer resistance. A significant QTL × sex interaction was found for lung tumor phenotype: separate analyses of females and males revealed clear linkage to chromosome 1 in males and almost no linkage in females. In contrast, the skin tumor locus had no apparent QTL × sex interaction. Besides the *Lsktm1* locus on chromosome 1, we found no further evidence for loci reaching the statistical threshold of genetic linkage for skin tumor multiplicity. In contrast, we identified an additional genetic linkage for lung tumorigenesis (as described in the section *Identification of* Par4 *locus linked to lung tumorigenesis*).

### Identification of *Par4* locus linked to lung tumorigenesis

**Figure 2:**
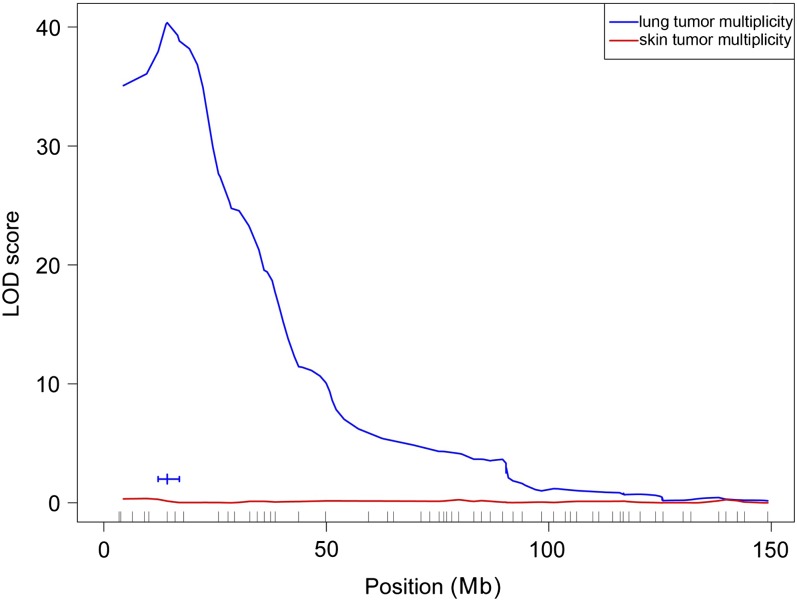
Interval mapping analysis of chromosome 6 region for skin and lung tumor susceptibility in SWR/J x (SWR/J x Car-R) mice. LOD curve for total lung tumor phenotype, with 1.5-LOD support interval indicated at the bottom. Map positions of SNP markers along the chromosome are indicated by small vertical lines.

Interval mapping analysis provided strong evidence for a QTL on chromosome 6 linked to lung tumor phenotype (Figure 2). The LOD score reached a peak of 40.4, near the marker rs3678711 at 14.24 Mb. The 1.5-LOD support interval spanned 5.9 Mb, from rs13478626 to rs13478641, in the region of the Pulmonary adenoma resistance 4 (*Par4*) locus, which had previously been mapped in F2 progeny obtained by crossing the lung-tumor-resistant BALB/cJ mouse with the lung-tumor-susceptible SWR/J mouse (Manenti *et al.* 1997). This QTL explained approximately 43% of the phenotypic variation in the backcross mice.

As observed for the chromosome 1 locus, heterozygous mice had fewer tumors than homozygotes, indicating that the Car-R alleles reduced the lung tumor multiplicity significantly. There was no evidence for additional lung tumor modifier loci, even after controlling for the loci on chromosomes 1 and 6.

### Search for candidate genes in the *Lsktm1* locus

**Figure 3:**
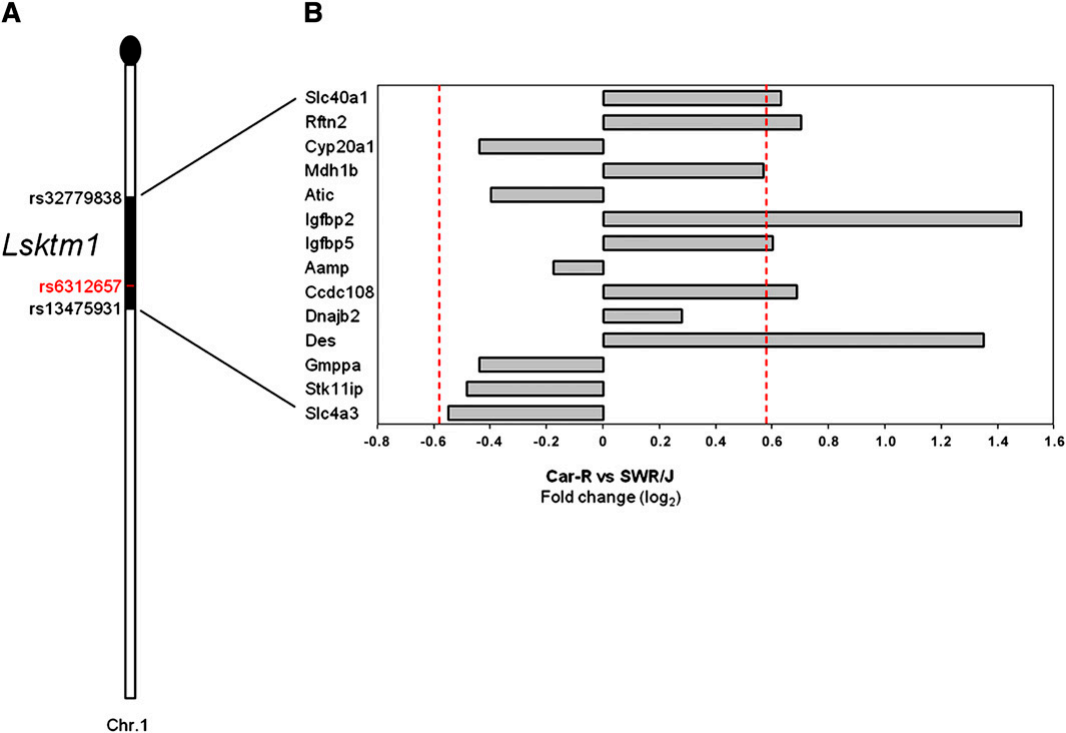
Differentially expressed genes in the *Lsktm1* locus. (A) Position of the *Lsktm1* interval on chromosome 1. The two markers delimiting the 1.5-LOD support interval of the locus, as well as the marker at the LOD score peak for lung tumor multiplicity (rs6312657, in red), are indicated. (B) Bar chart of expression differences (Car-R *vs.* SWR/J) for genes in proximity of the LOD peak expressed in lung with a significance of *P* < 0.001 and ordered by their physical position. Expression difference is represented as log_2_-transformed fold change between the mean expression scores of the two strains. The dashed lines across the bars identify genes with >1.5-fold different mRNA levels between the two mice strains.

Given the strong linkage of both *Lsktm1* and *Par4* loci to lung tumorigenesis, we analyzed gene expression in normal lung tissue of 15 Car-R and 13 SWR/J mice to identify candidate genes whose differential expression may account for the observed phenotypic variations. We first focused on the 1.5-LOD support interval of the LOD score peak for the lung tumor phenotype, from 41.15 Mb to 76.37 Mb, corresponding to markers rs32779838 and rs13475931. According to the Ensembl database (www.ensembl.org/Mus_musculus), this region contains 230 known genes. From expression microarray analysis, 92 of these genes were expressed in the lung tissue of both Car-R and SWR/J mice and mapped in the *Lsktm1* locus. Of these genes, 14 were differentially expressed between Car-R and SWR/J mice at nominal *P* < 0.001 (Figure 3). However, only six genes, namely *Slc40a1*, *Rftn2*, *Igfbp2*, *Igfbp5*, *Ccdc108*, and *Des*, showed >1.5-fold different mRNA levels between the two strains: all were upregulated in Car-R mice.

**Figure 4:**
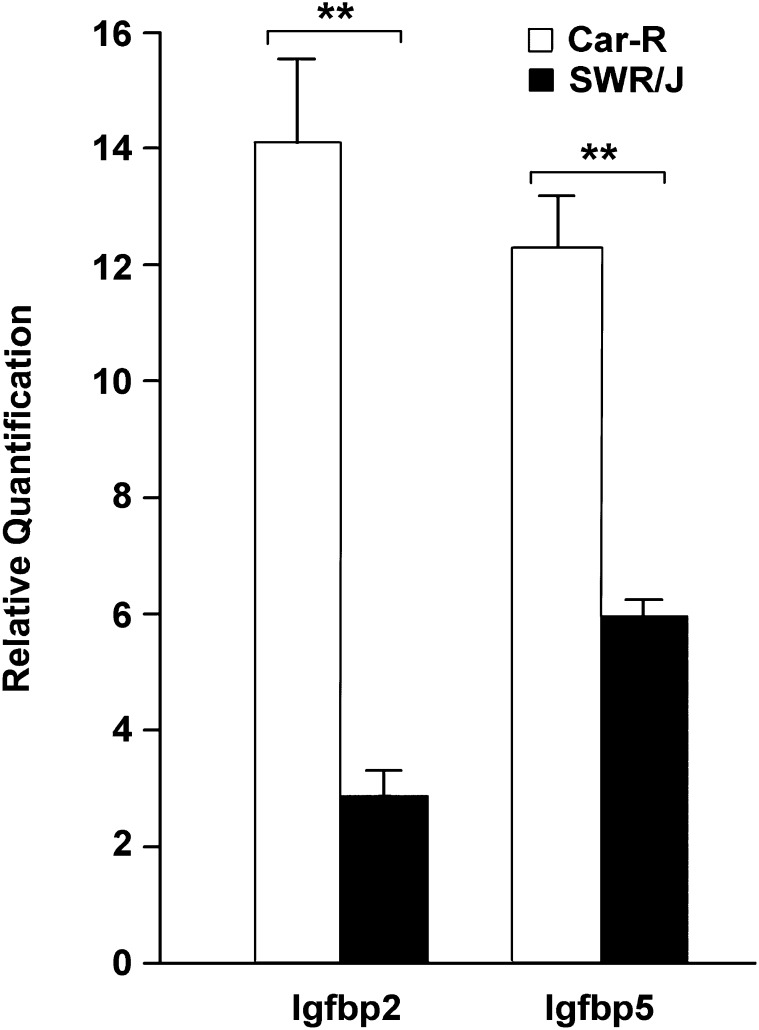
Expression analyses of *Igfbp2* and *Igfbp5* genes in normal lung tissue by qPCR. Transcript levels of SWR/J (n = 13) were compared with those of Car-R (n = 15). Relative quantification represents normalized expression units assessed using the comparative cycle threshold method and a pool of tissues RNA as calibrator. Error bars represent SEM. ^**^P < 0.001, ANOVA.

We were particularly intrigued by *Igfbp2* and *Igfbp5*, which are closely located in a tail-to-tail orientation. These genes belong to the insulin-like growth factor binding protein (IGFBP) superfamily and are known to influence tumorigenesis in humans (Hung *et al.* 2008; Rho *et al.* 2008; Diehl *et al.* 2009). Analysis of *Igfbp2* and *Igfbp5* genes by qPCR in the same Car-R and SWR/J mice confirmed the increased expression in normal lung tissue of Car-R mice, respectively about 5- and 2-fold greater (*P* < 0.001 for either genes, ANOVA; Figure 4).

We repeated the analysis searching for genes that were differentially expressed in lung between Car-R and SWR/J mice in the 1.5-LOD support interval of the *Par4* locus. This region contains 15 genes, of which 7 were found to be expressed in lung tissue. However, no gene had a differential expression exceeding 1.5-fold between the two murine lines.

### Overexpression of *Igfbp5* or *Igfbp2* inhibits clonogenicity of lung cancer cell lines

**Figure 5:**
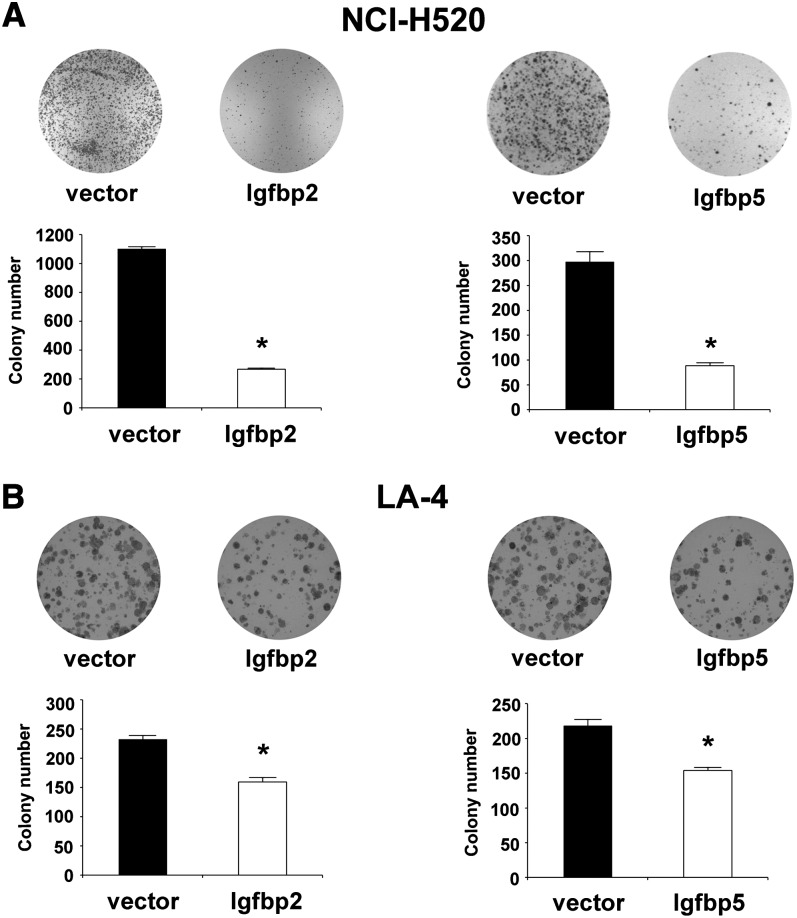
Clonogenic assay of *Igfbp2* and *Igfbp5* genes in two lung cancer cell lines. (A) Inhibition of *in vitro* colony formation of human lung carcinoma NCI-H520 cells by transfection with vectors encoding *Igfbp2* (left) and *Igfbp5* (right) compared with empty vectors. (B) Inhibition of *in vitro* colony of mouse lung adenoma LA-4 cells by transfection with vectors encoding *Igfbp2* (left) and *Igfbp5* (right) compared with empty vectors. Data are mean and SE of 6 independent replicas. ^*^P < 0.0001, ANOVA.

Because the lung cancer-resistant Car-R mouse has greater pulmonary expression of *Igfbp5* and of *Igfbp2* than the cancer-susceptible SWR/J strain, we hypothesized that overexpression of these genes may suppress lung tumorigenesis. We therefore tested the effects of *Igfbp5* and *Igfbp2* overexpression on clonogenicity of lung cancer lines. Human squamous cell carcinoma NCI-H520 and murine lung adenoma LA-4 cell lines were transfected with vectors encoding murine *Igfbp5* or *Igfbp2* and, after selection with blasticidin, were scored for colony formation. The expression of either recombinant proteins was confirmed by Western blotting analysis using anti-V5-HRP antibody (not shown). Overexpression in human NCI-H520 cells had a significant inhibitory effect on colony growth, decreasing the number of colonies by 70% and 76%, for *Igfbp5* and *Igfbp2*, respectively, compared with mock-transfected cells (*P* < 0.0001, ANOVA; Figure 5A). In the murine LA-4 cell line, colony formation reduced by 30% and 31%, for *Igfbp5* and *Igfbp2*, respectively, (*P* < 0.0001, ANOVA; Figure 5B). These data support a role of *Igfbp5* and *Igfbp2* as lung tumor suppressors.

## Discussion

In this study, genome-wide analysis of SNPs in SWR/J x (SWR/J x Car-R) backcross mice was carried out to identify cancer modifier loci involved in both skin and lung tumorigenesis. We identified a single novel locus, called *Lsktm1*, affecting skin and lung tumor susceptibility on chromosome 1. This finding confirms our hypothesis that a subset of Car-R−derived cancer-resistance alleles may control two cancer phenotypes in the backcross population.

A strong lung tumor modifier locus was also found on chromosome 6, in the same region of *Par4* locus (Manenti *et al.* 1997). The reason why this locus, affecting only lung tumorigenesis, segregated in Car-R mice is not clear, although the high frequency of the resistance allele in inbred mouse strains (Zaffaroni *et al.* 2005) may provide an explanation. In our previous work, sequence analysis for candidate genes in the *Par4* locus pointed to *Met*, which carried a nonconservative Arg968Cys change between the lung tumor−resistant BALB/cJ strain and the lung tumor−susceptible SWR/J strain (Zaffaroni *et al.* 2005). Because in our backcross population the LOD score peak for lung tumor linkage also was close to the *Met* gene, these findings confirm the involvement of this gene in lung tumor predisposition. Although we cannot exclude the presence of additional cancer modifier genes mapping close to the *Met* gene, gene expression analysis in normal lung tissue did not reveal any genes that were differentially expressed between Car-R and SWR/J mice in the *Par4* locus region.

To identify candidate cancer modifier genes in the *Lsktm1* locus, we analyzed the pulmonary expression of genes residing near the LOD score peak, assuming that the SNPs associated with lung tumor multiplicity can regulate in *cis* the expression of *Lsktm1* genes, as has been shown for loci associated with other diseases (Libioulle *et al.* 2007; Moffatt *et al.* 2007). We identified six transcripts that were significantly greater in normal lung tissue of Car-R than SWR/J mice. Two of these genes, namely *Igfbp2* and *Igfbp5*, are important components of the insulin-like growth factor (IGF) axis, which plays a role in controlling cell survival, differentiation, and apoptosis by enhancing or inhibiting the activity of IGFs in a cell- and tissue-specific manner (Schneider *et al.* 2002; Beattie *et al.* 2006; Tripathi *et al.* 2009).

Overexpression of either *Igfbp5* or *Igfbp2* in two lung cancer cell lines inhibited clonogenicity, suggesting that their upregulation *in vivo* may protect from tumor predisposition. The candidacies of *Igfbp5* and *Igfbp2* as tumor suppressors also are consistent with the observed sex modulation of lung tumor multiplicity phenotype in the backcross population and with the significant interaction between the chromosomal region, where these genes map, and sex status in the control of lung tumor multiplicity. Indeed, both *Igfbp5* and *Igfbp2* are modulated by sex hormones (Gregory *et al.* 1999; Rooman *et al.* 2005; Kamangar *et al.* 2006) and may have gender-related effects (Salih *et al.* 2005). In humans, overexpression of *IGFBP5* inhibited *in vitro* cell proliferation or angiogenesis in different tumor types (Hung *et al.* 2008; Rho *et al.* 2008; Su *et al.* 2011), whereas low serum *IGFBP5* levels correlated with poor prognosis of lung cancer patients (Shersher *et al.* 2011). Interestingly, population-based association studies in humans detected a significant association between cancer risk and SNPs mapping in a 50-kb interval encompassing the *IGFBP5* and *IGFBP2* genes (Garner *et al.* 2008; Niu *et al.* 2011). These findings suggest that genetic variations in the genomic region containing these genes may also modulate risk of cancer in humans.

Other candidate genes, such as *Des*, *Ccdc108*, *Slc40a1*, or *Rftn2*, also found in the *Lsktm1* locus, might act synergistically with *Igfbp* genes to confer the tumor phenotype. If this scenario is found to be true, then it could be possible that distinct genes in the *Lsktm1* locus have specific roles in the predisposition to lung or skin tumor susceptibility. In addition, the fact that we found only one locus associated with skin tumorigenesis suggests that larger genetic crosses are necessary to reach a sufficient detection power for mapping skin tumor modifier loci with small effects that have segregated in the Car-R mouse model.

In conclusion, genome-wide analysis of genetic markers in a backcross population allowed the identification of a new tumor modifier locus (*Lsktm1*) involved in modulating susceptibility to skin and lung tumorigenesis. Within this locus, *Igfbp5* and *Igfbp2* are good candidates for cancer modifier of lung tumorigenesis. Identification of mouse cancer modifier genes is a step toward understanding the genetic and biochemical mechanisms of inherited cancer predisposition. This knowledge can be translated to humans, where homologous cancer modifier genes can be the target for the development of diagnostic, preventive, and therapeutic strategies against cancer.
